# Novel Function of Lysine Methyltransferase G9a in the Regulation of Sox2 Protein Stability

**DOI:** 10.1371/journal.pone.0141118

**Published:** 2015-10-22

**Authors:** Jae-Young Lee, Se-Hwan Lee, Sun-Hee Heo, Kwang-Soo Kim, Changhoon Kim, Dae-Kwan Kim, Jeong-Jae Ko, Kyung-Soon Park

**Affiliations:** 1 Department of Biomedical Science, College of Life Science, CHA University, Seoul, Korea; 2 Department of Biomedical Science, Graduate School of Biomedical Science & Engineering, Hanyang University, Seoul, Korea; St. Georges University of London, UNITED KINGDOM

## Abstract

G9a is a lysine methyltransferase (KMTase) for histone H3 lysine 9 that plays critical roles in a number of biological processes. Emerging evidence suggests that aberrant expression of G9a contributes to tumor metastasis and maintenance of a malignant phenotype in cancer by inducing epigenetic silencing of tumor suppressor genes. Here, we show that G9a regulates Sox2 protein stability in breast cancer cells. When G9a lysine methyltransferase activity was chemically inhibited in the ER(+) breast cancer cell line MCF7, Sox2 protein levels were decreased. In addition, ectopic overexpression of G9a induced accumulation of Sox2. Changes in cell migration, invasion, and mammosphere formation by MCF7 cells were correlated with the activity or expression level of G9a. Ectopic expression of G9a also increased Sox2 protein levels in another ER(+) breast cancer cell line, ZR-75-1, whereas it did not affect Sox2 expression in MDA-MB-231 cells, an ER(-) breast cancer cell line, or in glioblastoma cell lines. Furthermore, treatment of mouse embryonic stem cells with a KMT inhibitor, BIX-01294, resulted in a rapid reduction in Sox2 protein expression despite increased Sox2 transcript levels. This finding suggests that G9a has a novel function in the regulation of Sox2 protein stability in a cell type-dependent manner.

## Introduction

Lysine methyltransferase G9a is ubiquitously expressed in most tissues, including bone marrow, thymus, spleen, lymph node, and fetal liver [1]. G9a knock-out mice are embryonic lethal between embryonic (E) days E9.5–E12.5. Even though G9a-/- embryonic stem cells (ESCs) do not show abnormalities in culture, they exhibit severe differentiation defects, suggesting a role for G9a in lineage commitment and differentiation [2]. Consistent with this notion, there is considerable evidence that G9a represses Oct3/4, Nanog, and DNMT3L, which are required for maintenance of pluripotent differentiation potential in ESCs [2]. G9a is also implicated in genomic imprinting. G9a is recruited by the non-coding RNAs *Air* and *Kcnqlot1* to target genes and stimulate the formation of heterochromatin in a lineage-specific manner [3,4].

G9a localizes to euchromatin in a heteromeric complex with a G9a-like protein (GLP), a highly homologous lysine methyltransferase, to repress gene transcription, especially during embryonic development. G9a-mediated gene repression is associated with its ability to mono- and dimethylate H3K9 and H3K27 [2,5–10]. Additionally, G9a/GLP can directly recruit DNA methyltransferases to promoters, resulting in gene repression via the methylation of CpG islands [11,12]. In addition to the methylation of histone 3, G9a mediates methylation of various non-histone proteins, including p53 [6,13].

While substantial studies suggest that the function of G9a is associated with transcriptional repression, it switches from a repressor to an activator by changing its interacting partners. For example, G9a interacts with the H3K4 demethylase Jarid1a at the embryonic E^y^ globin promoter to repress its expression, while it recruits Mediator to the β^maj^ promoter, resulting in its activation [14]. Similarly, G9a also acts with CARM1 and p300 as a co-activator of nuclear receptors, independent of its lysine methyltransferase activity [15].

A large number of studies have indicated that G9a is also highly expressed in a variety of human cancers, such as breast, lung, and hepatocellular carcinoma [5,16,17]. Knockdown of G9a in lung cancer cell lines causes apoptosis and growth arrest with an increase in the sub-G1 population [18]. In prostate cancer, downregulation of G9a results in centrosome disruption, inhibition of cell growth, and increased cellular senescence in cancer cells [19]. Studies in aggressive forms of lung cancer further revealed that G9a expression is correlated with poor prognosis, increased cell migration, invasion, and metastasis [5]. However, the molecular basis of G9a activity in cancer cells is not well understood.

Here, we identify an essential role for G9a in maintaining Sox2 protein stability in ER(+) breast cancer cell lines. We found that lysine methyltransferase activity is closely related to the accumulation of Sox2 protein, the protein level of which is closely related to cell migration, invasion, and mammosphere formation by ER(+) breast cancer cell lines. However, G9a did not affect Sox2 protein levels in MDA-MB-231 cells, an ER(-) breast cancer cell line, or in glioblastoma cell lines. These findings provide evidence that G9a-mediated stabilization of the Sox2 protein depends on the subcellular context of each cell.

## Materials and Methods

### Cell culture

The cell lines MCF-7 and the mouse embryonic stem cell line J1 were obtained from the American Type Culture Collection (ATCC, USA). MCF-7 cells were cultured with 10% fetal bovine serum and 1% penicillin/streptomycin in Dulbecco’s modified Eagle’s medium (DMEM). Mouse embryonic stem cells (mESCs) were maintained on 0.1% gelatin coated dishes in DMEM (Gibco Invitrogen, Carlsbad, CA, http://www.invitrogen.com) supplemented with 10% horse serum (Gibco Invitrogen), 2 mM glutamine, 100 U/ml penicillin, 100 μg/ml streptomycin (Gibco Invitrogen), 1x non-essential amino acids (Life Technologies), 0.1 mM 2-mercaptoethanol (Sigma-Aldrich, St Louis, MI, http://www.sigmaaldrich.com), and 1,000 U/ml LIF (Chemicon, Temecula, CA, http://www.chemicon.com). To induce sphere formation, MCF7 cells were detached from the culture dishes using 0.05% Trypsin-EDTA solution and then suspended in non-coated Petri dishes. Cells were grown in serum-free DMEM/F12 medium containing B27 (10889–088, Invitrogen), 20 ng/ml epidermal growth factor, 20 ng/ml basic fibroblast growth factor (13256–029, Invitrogen), and 20 ng/ml insulin growth factor 1 (291-G1, R&D Systems). Mammospheres were cultured for 5–7 days.

### Plasmid, siRNA, and transfection

Human G9a, Sox2 expression plasmids were purchased from Addgene (Cambridge, MA) and human G9a siRNAs (L-006937-00-0010) and Sox2 siRNAs (L-011778-00-0005) were obtained from Dharmacon, Inc (Chicago, IL). Plasmids or siRNAs were transfected using Lipofectamine 2000 (Invitrogen) according to the manufacturer’s instructions. Cells were collected 24 hr after transfection for further analysis.

### RNA isolation and real-time PCR

Total cellular RNA was extracted using TRIzol reagent (Invitrogen), according to the manufacturer’s instructions. Total RNA (2 μg) was used for single-stranded cDNA synthesis using Omniscript Reverse Transcriptase (Qiagen, Hilden, Germany). Gene-specific primers were designed for Sox2, Oct4, G9a, and GAPDH, as described in S1 Table. Quantitative real-time PCR was performed with the CFX96 Touch^™^ Real-Time PCR Detection System (Bio-Rad Laboratories, CA, USA) using SYBR Green I (Qiagen, Valencia, CA, USA). Cycling conditions were as follows: 40 cycles of 95°C for 30 s, 49°C for 30 s, and 72°C for 30 s. Results were calculated as relative expression and normalized to internal human GAPDH using the ΔC_t_ method.

### Protein extraction and Western blotting

Total protein was isolated using cell lysis buffer (#9803, Cell Signaling Technology, Inc.) according to the manufacturer’s instructions. Proteins (20 μg) were separated by 10% SDS-PAGE and transferred to a polyvinylidene difluoride (PVDF) membrane (Millipore, Bedford, MA). The membrane was incubated with antibodies against Sox2 (sc-20088, Santa Cruz Biotechnology, Inc.), Oct4 (sc-5279, Santa Cruz Biotechnology, Inc.), G9a (#3306, Cell Signaling Technology, Inc.), and β-actin (sc-47778, Santa Cruz Biotechnology, Inc.) overnight at 4°C, followed by incubation with secondary antibody for 1 h at room temperature. Immunoreactive proteins were detected using the WEST-ZOL^®^ (plus) Western Blot Detection System (iNtRON, Korea).

### Migration and invasion assay

Migration of MCF7 cells was assessed by scratch/wound assays. Monolayers formed by cells were wounded using a micropipette tip when the cells were fully confluent. Detached cells were removed by washing with medium and plates were photographed at the indicated times. The Matrigel invasion assay was performed using 24-well Transwell inserts (6.5 mm diameter, 8 μm pores, Corning, NY, USA) coated with 30 μg of Matrigel (#356231, BD Biosciences). Cells (1 x 10^5^) were seeded in serum-free medium in the upper chamber and DMEM containing 10% FBS was added to the lower chamber. Cells were incubated to assess invasion through the membrane for 48 h. Invasive cells were fixed and stained with Crystal violet (Sigma). The intensity of the Crystal violet staining was measured using GelQuant software (biochemlabsolutions.com) and expressed as the means ± SE of triplicate wells.

### Co-immunoprecipitation (Co-IP)

Cells were lysed by sonication in Co-IP buffer (20 mM Tris-HCl, 137 mM NaCl, 1% Triton X-100, 2 mM EDTA) containing Protease and Phosphatase Inhibitor Cocktail (Pierce Biotechnology, Rockford, IL, USA), and then centrifuged at 12,000 × g for 15 min at 4°C. Following protein isolation, 500 μg of total protein per sample were diluted with Co-IP buffer, pre-cleared with normal goat IgG and protein A/G-plus beads (Santa Cruz Biotechnology, Santa Cruz, CA, USA) and then immunoprecipitated with 2 ug of anti-Sox2 (sc-20088, Santa Cruz Biotechnology, Inc.) or 4 ug of anti-G9a antibody (#3306, Cell Signaling Technology, Inc.) overnight at 4°C. After washing, lysate/beads were boiled in 20 μL of SDS sample buffer, and a 10 μL aliquot of the eluate was fractionated by 12% SDS—PAGE followed by Western blotting to confirm physical interaction between Sox2 and G9a. Reactive bands were detected with an ECL system (Amersham, Amersham, UK) according to the manufacturer’s instructions.

### Statistical analysis

The data are presented as the means ± SE. The statistical significance of the results was assessed using one-way ANOVA, followed by Student’s t-test.

## Results

### Chemical inhibition of G9a activity decreases Sox2 protein stability in breast cancer cells

**Fig 1:**
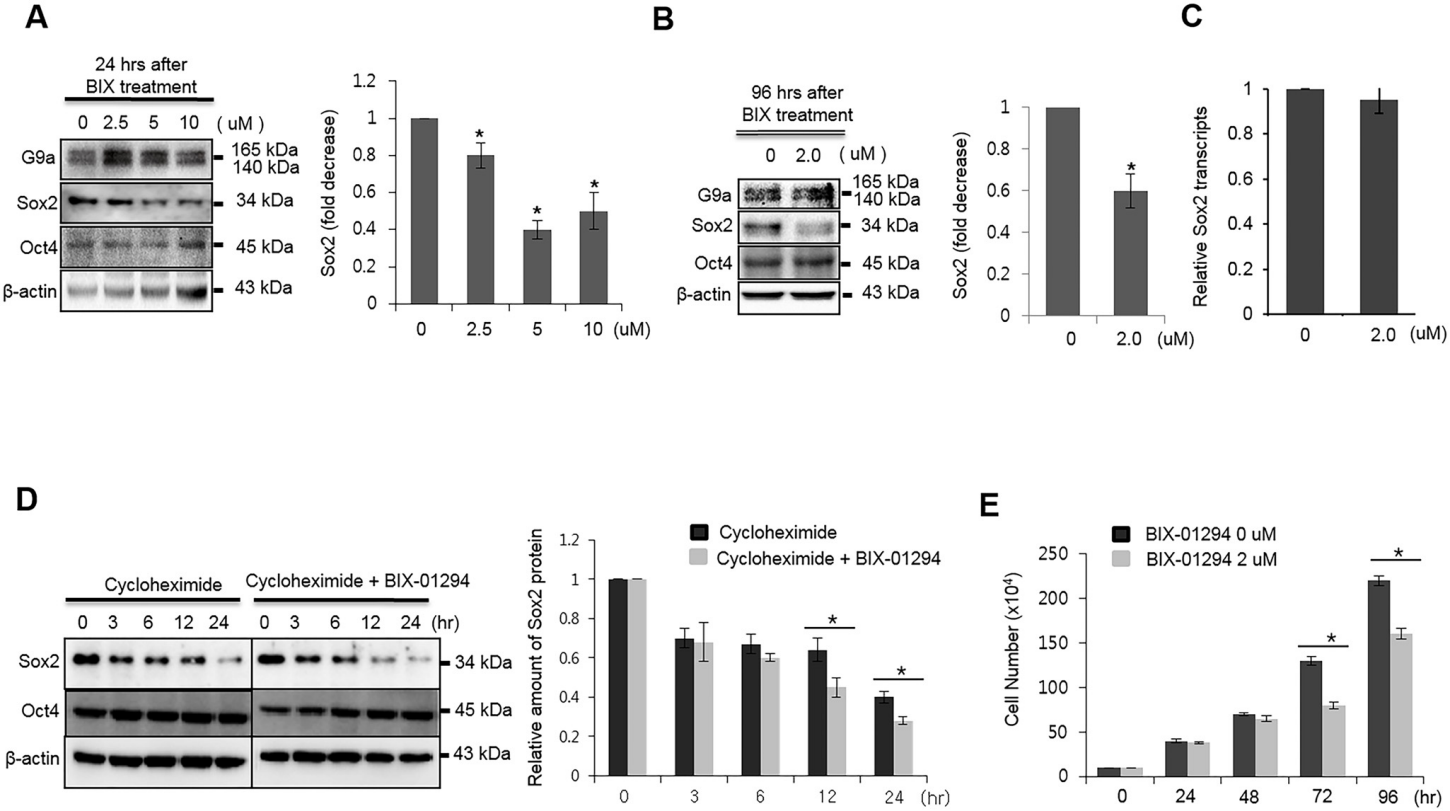
Chemical inhibition of G9a activity reduces the stability of Sox2 protein in MCF7 cells. (A) MCF7 cells were treated with the indicated doses of BIX-01294 for 24 h. Whole cell lysates were harvested and analyzed by immunoblotting with G9a-, Sox2-, and Oct4-specific antibodies. (B) MCF7 cells were treated with 0.2 μM of BIX-01294 for 96 h, and whole cell lysates were analyzed for the expression of Sox2 and Oct4. (C) MCF7 cells were treated with 2.0 μM BIX-01294 for 96 h, and the levels of Sox2 transcripts were analyzed by real-time RT-PCR analysis. mRNA levels were normalized to those of GAPDH. (D) Sox2 and Oct4 protein levels in MCF7 cells were analyzed by immunoblotting after cells were treated with 2.0 μM BIX-01294 in the presence of the protein synthesis inhibitor, cycloheximide (5 μM). The graph shows band intensity data normalized according to the controls and β-actin. (E) Cell growth was analyzed by counting cell numbers at the indicated times after treatment with 2.0 μM BIX-01294. For quantitative analysis of the immunoblotting results, the mean density of the Sox2 band was measured with Multi Gauge V3.0 software, and the band density was divided by the density of β-actin to obtain the normalized band density. * P < 0.05 compared with the control group. Data are expressed as the mean ± SD and are representative of three independent experiments.

The expression of lysine methyltransferase G9a is highly correlated with metastasis of various cancers and a poor prognosis [20]. Since monomethylated Sox2 interacts with the E3 ligase WWP2 in ESCs, thereby causing its ubiquitination and degradation [21], we questioned whether the activity of G9a is related to the protein stability of Sox2 in breast cancer. As a first step, we examined whether Sox2 protein stability is regulated by G9a activity. Unexpectedly, chemical inhibition of G9a activity using the KMT inhibitor BIX-01294 resulted in reduced amounts of Sox2 protein in MCF7 breast cancer cells (Fig 1A). By contrast, the levels of Oct4 proteins were unchanged. Sox2 protein levels were also decreased when cells were treated with low concentrations of BIX-01294 for 96 h (Fig 1B). In contrast to the changes observed in protein levels, transcript levels of Sox2 were not changed by treatment with BIX-01294 (Fig 1C). The transcription of Sox2 in MCF7 cells was not affected by higher concentrations of BIX-01294 (S1 Fig). When Sox2 protein biosynthesis was blocked by treatment with cycloheximide, BIX-01294 treatment induced degradation of Sox2 (Fig 1D). The proliferation rate of MCF7 cells also decreased upon BIX-01294 treatment (Fig 1E). When we treated MCF-7 cells with another inhibitor of G9a, UNC0638, we obtained similar results to those obtained with BIX-01294, i.e., the amount of Sox2 protein decreased upon treatment with 10 μM UNC0638 (S2 Fig). However, chemical inhibition of G9a activity or siRNA-mediated inhibition of G9a expression had no effect on Sox2 protein stability in ER(-) MDA-MB-231 cells (S3 Fig). Notably, G9a was expressed at high levels in ER(+) MCF7 and ZR-75-1 cells, but was hardly detectable in ER(-) MDA-MB-231 cells (S4 Fig). These results indicate that the stability of the Sox2 protein was affected by G9a lysine methyltransferase activity only in ER(+) MCF7 cells.

### Sox2 protein stability is affected by G9a expression

**Fig 2:**
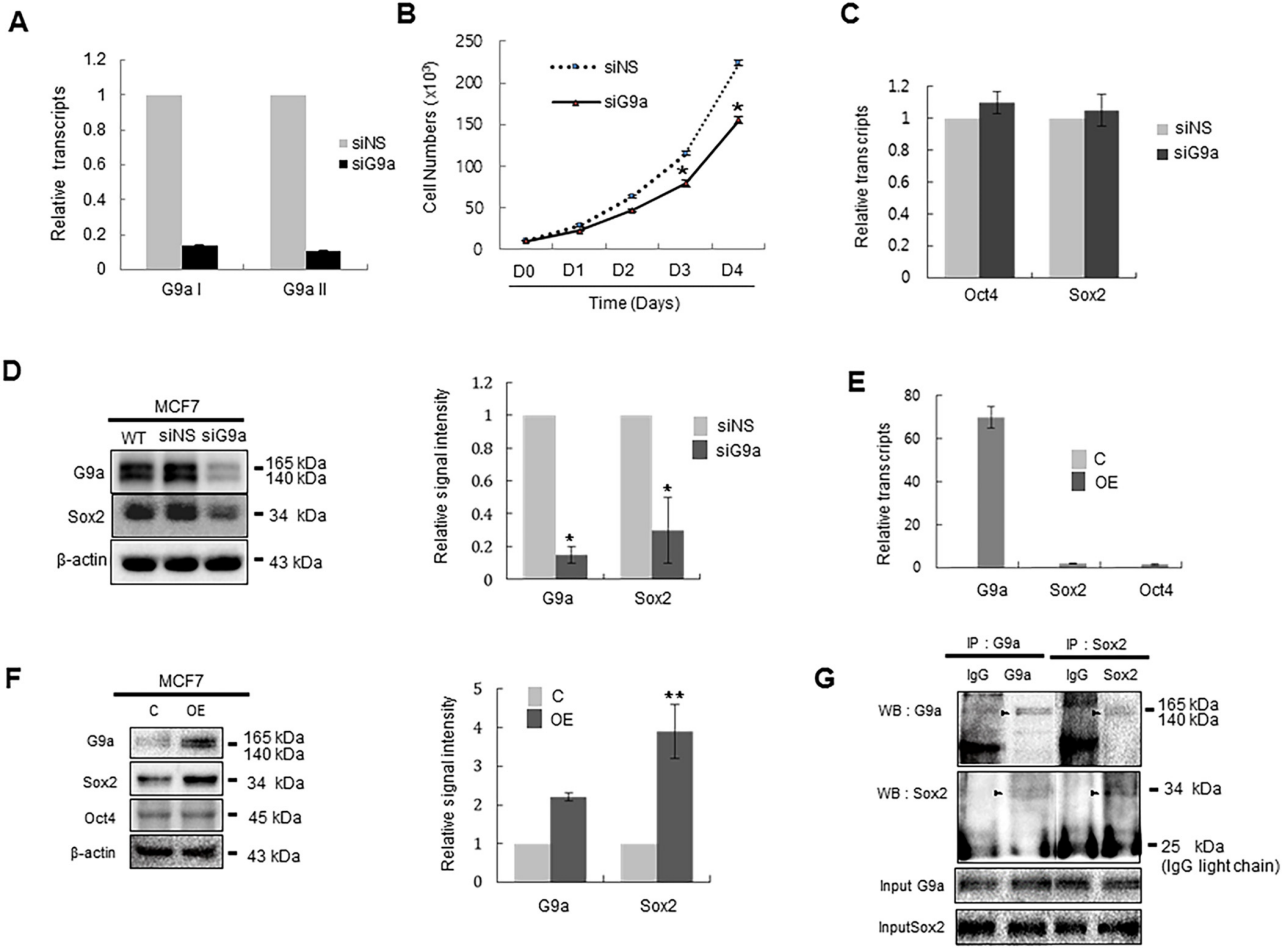
The expression level of G9a affects the amount of Sox2 protein in MCF7 cells. (A) MCF7 cells were transfected with siNS or siG9a for 24 h, and G9a transcript levels were analyzed by real-time PCR by priming two positions of G9a ORF (G9aI and G9aII). mRNA levels were normalized to those of GAPDH. G9aI and G9a2 primer sets were designed to amplify 1299~1531 bp and 2326~2637 bp of G9a ORF, respectively. (B) The effect of G9a on the proliferation of MCF7 cells. MCF7 cells (1 × 10^4^) were transfected with siNS or siG9a and then counted at the indicated times post-transfection. (C) MCF-7 cells were transfected with siNS or siG9a for 24 h, and the effect of G9a knockdown on the transcription of Oct4 and Sox2 was quantitatively analyzed by real-time RT-PCR. (D) MCF-7 cells were transfected with siNS or siG9a for 24 h, and the effect of G9a knockdown on the amount of Oct4 and Sox2 protein was analyzed by immunoblot analysis. (E) MCF-7 cells were transfected with control or G9a expression plasmids for 24 h, and the effect of G9a overexpression on the transcription of Sox2 and Oct4 was quantitatively analyzed by real-time RT-PCR. (F) MCF-7 cells were transfected with control or G9a expression plasmids for 24 h, and the effect of G9a overexpression on the amount of Sox2 and Oct4 was quantitatively analyzed by immunoblot analysis. (G) Co-immunoprecipitation of endogenous Sox2 and G9a in MCF7 cells. Total protein extract of MCF7 cells was immunoprecipitated with Sox2 and then immunoblotted with G9a antibody. The reciprocal experiment was also performed in which total protein of MCF7 cells was immunoprecipitated with G9a and then immunoblotted with Sox2 antibody. The specific bands corresponding to the G9a and Sox2 was arrowed. Abbreviations: siNS, non-specific siRNA; siG9a, siRNA targeting G9a; C, control plasmid; OE, G9a-expressing plasmid; IP, immunoprecipitation; IB: immunoblotting. For quantitative analysis of the immunoblotting results, the mean density of the Sox2 or G9a bands was measured with Multi Gauge V3.0 software. The band density was then divided by the density of β-actin to obtain the normalized band density. *P < 0.05 and **P < 0.01 compared with the control group. Data are expressed as the mean ± SDS and are representative of three independent experiments.

We next tested whether the level of G9a expression correlates with Sox2 protein stability. To examine this, we transiently knocked-down G9a expression by siRNA transfection (Fig 2A). Similar to the results observed following BIX-01294 treatment, the rate of cell proliferation of MCF7 cells was decreased by transfection of siG9a (Fig 2B).

Consistent with the results obtained after BIX-01294 treatment, suppressing G9a expression did not alter the transcription of Sox2 or Oct4 in MCF7 cells (Fig 2C). However, transfection of siG9a led to a significant reduction in Sox2 protein levels, even though it did not affect Oct4 protein levels (Fig 2D). Conversely, ectopic expression of G9a increased the amount of Sox2 protein independent of Sox2 transcription even when the transfection efficiency of G9a expressing plasmid into MCF7 cells is about 30% (Fig 2E and 2F, S5 Fig). The growth rate of MCF7 cells also increased following ectopic expression of G9a (S6 Fig). To determine whether G9a interacts with Sox2 in MCF7 cells, immunoprecipitation was performed using non-specific IgG or an anti-Sox2 antibody. Immunoblot analysis of the precipitates with antibody against G9a showed that G9a was recovered in the Sox2 immmunoprecipitates, and reciprocal immunoprecipitation with anti-G9a antibodies confirmed this interaction (Fig 2G). These data indicate that G9a increases Sox2 protein stability in MCF7 breast cancer cells, possibly through direct interaction.

### G9a regulates the oncogenic characteristics of MCF7 cells

**Fig 3:**
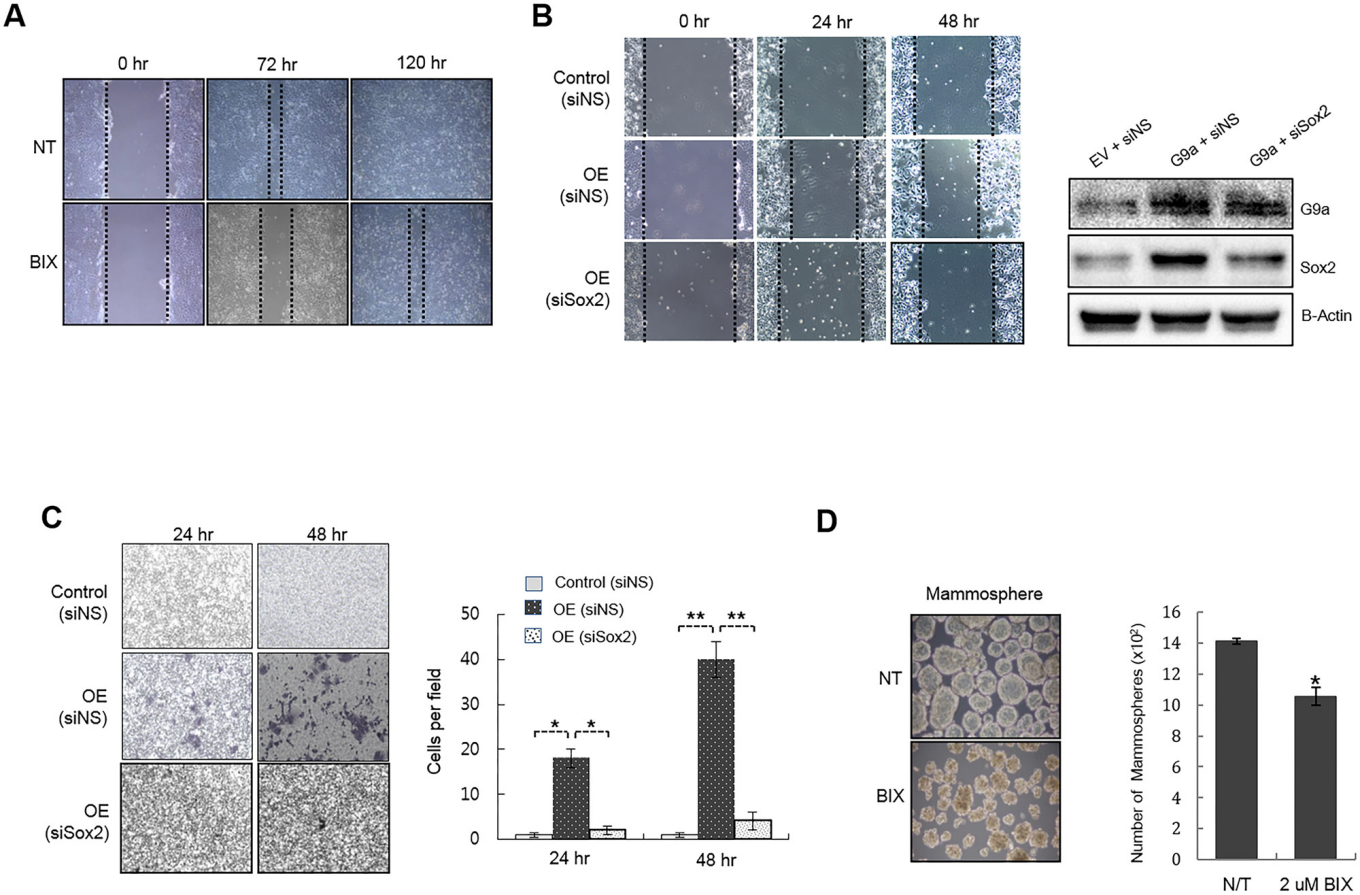
Expression level of G9a is correlated with the migration and invasion of MCF7 cells. (A) Wound-healing assay examining the effect of chemical inhibition of G9a activity on the migration of MCF7 cells. MCF7 cells were treated with 0.2 μM BIX-01294 and wounded using a micropipette tip when the cells were fully confluent. Cell migration was then monitored. (B) Wound-healing assay to examine the effect of ectopic expression of G9a on the migration of MCF7 cells. MCF7 cells were transfected with control or G9a expression plasmids and then re-seeded for the migration assay. When fully confluent, the cells were wounded using a micropipette tip, and cell migration was photographed at the indicated times. (C) The effect of G9a on the invasiveness of MCF7 cells was examined in a Transwell invasion assay. MCF7 cells were first transfected with control or G9a expression plasmids along with siNS or siSox2. Twenty-four hours later, cells were suspended in serum-free medium and seeded in 24-well Matrigel-coated Transwell chambers. Cells crossing the Matrigel-coated filter were fixed and stained with Crystal violet. The number of stained cells was then counted. Representative pictures are shown (left panel). The average number of stained cells in five random microscopic fields from three independent experiments is presented graphically (right panel). (D) The effect of G9a on the mammosphere-forming ability of MCF7 cells was examined in cells maintained under low-serum non-adherent culture conditions for 7 days in the presence or absence of 2 μM BIX-01294. Abbreviations: NT, no treatment; Control (siNS), cells transfected with a control plasmid and non-specific siRNA; OE (siNS), cells transfected with a G9a-expressing plasmid and non-specific siRNA; OE (siSox2), cells transfected with a G9a-expressing plasmid and siSox2. *P < 0.05 and **P < 0.01 compared with the control group. Data are expressed as the mean ± SD and representative of three independent experiments.

Since Sox2 is a key player in the oncogenicity of breast cancer, we hypothesized that alteration of G9a activity would have an effect on the oncogenic characteristics of MCF7 cells, such as migration, invasion, and cancer stem cell characteristics. As expected, migration assay revealed that chemical inhibition of the lysine methyltransferase activity of G9a resulted in decreased cell migration compared with untreated cells (Fig 3A). Consistent with this, overexpression of G9a led to a marked increase in the migration of MCF7 cells (Fig 3B). siRNA-mediated suppression of Sox2 abrogated the effect of G9a on cell migration, indicating that overexpression of G9a stimulates the migration of MCF7 cells in a Sox-dependent manner. Next, we determined whether G9a overexpression affected the invasive capacity of MCF7 cells in a Matrigel assay. As expected, MCF7 cells transfected with G9a-expressing plasmids showed increased invasiveness; siSox2-mediated suppression of Sox2 abrogated this effect (Fig 3C). Because Sox2 plays a crucial role in promoting the stem cell characteristics of breast cancer cells, we examined whether G9a activity is related to the self-renewal of MCF7 cells by analyzing their ability to form mammospheres. When MCF7 cells were cultured under non-adherent conditions in the presence or absence of BIX-01294, cells treated with BIX-01294 formed fewer mammospheres, and those that were formed were smaller than those formed by untreated cells (Fig 3D). These results suggest that G9a plays an essential role in the oncogenic characteristics of MCF7 cells.

### G9a regulates Sox2 protein stability in ER(+) breast cancer cells and mouse embryonic stem cells, but not in ER(-) breast cancer cells and glioblastoma cells

**Fig 4:**
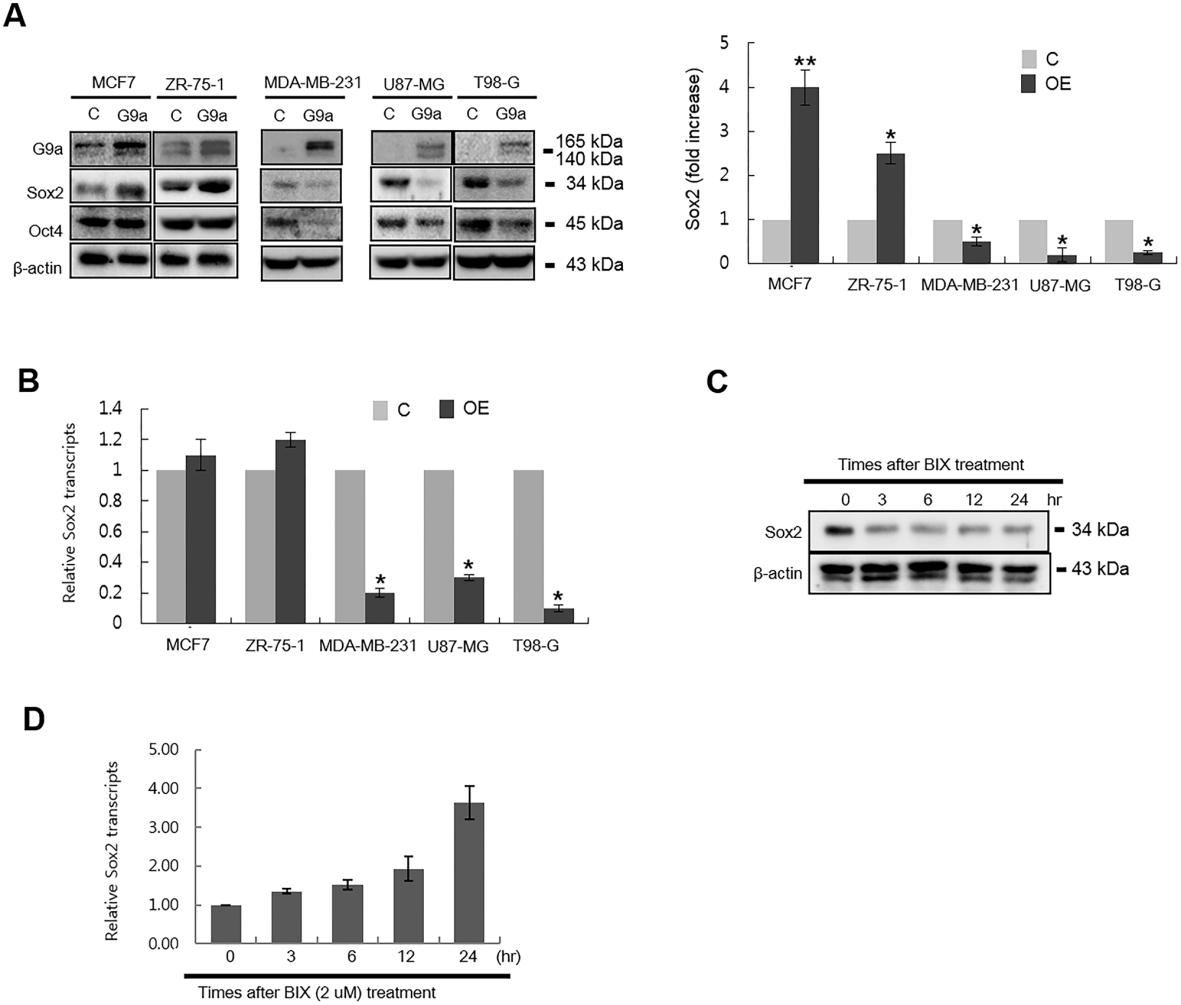
Effect of G9a on the transcription and protein expression of Sox2 in various cell types. (A) The effect of ectopic expression of G9a on Sox2 and Oct4 protein expression in three breast cancer cell lines (MCF7, ZR-75-1, and MDA-MB-231) and two glioblastoma cell lines (U87-MG and T98-G) was determined by immunoblot analysis. Each cell line was transfected with a G9a-expressing plasmid for 24 h and then harvested for analysis. For quantitative analysis of immunoblotting results, the mean density of the Sox2 band was measured with Multi Gauge V3.0 software, and the band density was divided by the density of β-actin to obtain the normalized band density. (B) Cells were transfected with control or G9a expressing plasmids for 24 h, and the effect of ectopic expression of G9a on the transcription of Sox2 was quantitatively analyzed by real-time RT-PCR. (C) The effect of G9a activity on Sox2 protein stability in mouse ESCs. Mouse ESCs were treated with 0.2 μM BIX-01294 for the indicated times and then harvested for immunoblot analysis. (D) The effect of G9a activity on the transcription of Sox2 in mouse ESCs. Mouse ESCs were treated with 0.2 μM BIX-01294 for the indicated times and then harvested for quantitative analysis of Sox2 transcription by real-time RT-PCR. Abbreviations: G9a, G9a-expressing plasmid; Sox2, Sox2-expressing plasmid; C, control plasmid. *P < 0.05 compared with the control group. Data are expressed as the mean ± SD and are representative of three independent experiments.

We next questioned whether G9a regulates Sox2 protein stability in other cell types. Interestingly, ectopic expression of G9a in ZR-75-1 cells, which like MCF7 cells are a representative ER(+) breast cancer cell line, increased Sox2 protein levels (Fig 4A). Contrary to the findings in MCF7 and ZR-75-1 cells, ectopic expression of G9a reduced Sox2 protein levels in MDA-MB-231 cells, an ER(-) breast cancer cell line, and in glioblastoma cells (U87-MG and T98-G; Fig 4A). Expression of Sox2 mRNA in ZR-75-1 cells was not affected by overexpression of G9a, suggesting that the mechanism by which ZR-75-1 regulates Sox2 levels upon G9a expression is similar to that in MCF-7 cells (Fig 4B). However, the transcription of Sox2 in MDA-MB-231, U87-MG, and T98-G was decreased by ectopic expression of G9a (Fig 4B). These results indicate that G9a regulates Sox2 expression in MDA-MB-231, U87-MG, and T98-G via epigenetic regulation of transcriptional expression rather than by modulating protein stability. Since Sox2 plays an essential role in maintaining pluripotency in ESCs, we next questioned whether treating mouse ESCs with a chemical inhibitor would affect G9a lysine methyltransferase-mediated Sox2 protein stability in a manner similar to that observed in MCF7 cells. Fig 4C shows that Sox2 protein levels in mouse ESCs decreased at 3 h after BIX-01249 treatment. We found it interesting that, in contrast to the changes observed in protein levels, Sox2 transcript levels were increased in a time-dependent manner (Fig 4D). Consistent with the increases of Sox2 transcript levels, we observed that the levels of H3K9me2 on the Sox2 promoter decreased significantly at 24 h after BIX-01294 treatment (data not shown). These results suggest that G9a regulates Sox2 levels in mouse ESCs through a dual mechanism: epigenetic regulation of transcription and regulation of protein stability. Taken together, we concluded that the ability of G9a to increase Sox2 protein stability depends on the intracellular environment of each cell.

## Discussion

Lysine methyltransferase G9a is a major histone modifier that links chromatin status to transcriptional gene regulation. Recent studies confirm that G9a methylates lysine residues within various non-histone proteins, including p53, Reptin, ACINUS, WIZ, CDYL1, and C/EBPβ [22–25]. Here, we provide evidence that G9a regulates Sox2 protein stability in ER(+) breast cancer cells and mouse ESCs.

First, chemical inhibition of G9a activity resulted in a decrease in Sox2 protein levels without any changes in Sox2 transcript levels. Second, siRNA-mediated suppression of G9a coincided with decreased levels of Sox2 protein expression without parallel changes in Sox2 transcripts. Third, overexpression of G9a in MCF7 and ZR-75-1 cells induced marked accumulation of Sox2 protein, even though it had no effect on Sox2 transcript levels.

We found it interesting that the effect of G9a overexpression on the Sox2 protein was only observed in ER(+) breast cancer cell lines such as MCF7 and ZR-75-1, but not in ER(-) MDA-MB-231 cells. This suggests the possibility that the role played by G9a in maintaining Sox2 protein stability is specific to ER(+) breast cancer cells. However, it is not clear how G9a act as a Sox2 protein stabilizer in ER(+) cells, or whether the effect of G9a on Sox2 protein stability depends on the ER signal. High expression of G9a in ER(+) MCF7 and ZR-75-1 cells is consistent with a previous report showing that G9a is a co-activator of nuclear receptors such as estrogen receptor α [26]. The expression pattern of G9a leads to the hypothesis that abundantly expressed G9a protein in ER(-) cells directly methylates Sox2 to protect it from ubiquitin-dependent degradation. Since G9a-mediated repression of E-cadherin in human breast cancer is mediated via interaction with Snail (which controls epithelial-mesenchymal transition during embryogenesis and tumor progression) [27], it is possible that G9a stabilizes the Sox2 protein through coordinated interaction with unidentified co-factors that are exclusively expressed by ER(+) breast cancer cells. The detailed mechanism(s) underlying the cell type-specific activity of G9a on Sox2 protein stability requires further investigation.

The role of G9a in regulating Sox2 protein stability in mouse ESCs is of particular interest because cancer cells share common characteristics with ESCs, i.e., cancer stem cells have the capacity for self-renewal [28]. It is intriguing that chemical inhibition of G9a rapidly reduced Sox2 protein levels and concomitantly increased the transcription of Sox2 in mouse ESCs (Fig 4C and 4D). Although the mechanism underlying this observation remains to be defined, this result suggests that G9a affects both promoter activity and Sox2 protein stability in mouse ESCs. It is possible that G9a functions epigenetically to regulate Sox2 promoter activity, while at the same time regulating Sox2 protein stability, possibly by protecting it from degradation. This dual activity of G9a in mouse ESCs may provide an advantage in that it optimizes the amount of Sox2 protein in mouse ESCs by finely tuning the transcriptional and post-translational mechanisms responsible for Sox2 expression.

We also found it interesting that, even though the effect of G9a on Sox2 protein expression in MCF7 and mouse ESCs is opposite to that in MDA-MB-231 cells, chemical inhibition of G9a activity led to a similar reduction in the proliferation of all three cell lines (S7 Fig). This suggests that the mechanism underlying G9a-mediated cell proliferation is independent of Sox2. The detailed mechanism by which G9a regulates cell proliferation requires further investigation.

The present study provides evidence that the amount of Sox2 protein in breast cancer cells is regulated by the expression and activity of G9a lysine methyltransferase. Therefore, it will be of particular importance to demonstrate that Sox2 is indeed methylated by G9a, and that methylated Sox2 is resistant to proteolytic degradation.

## Supporting Information

S1 Fig MCF-7 cells were treated with the indicated concentrations of BIX-01294, and the effect of various concentrations of BIX-01294 on the transcription of G9a, Sox2, and Oct4 was quantitatively analyzed by real-time RT-PCR. (TIF)

S2 Fig The effect of chemical G9a inhibitor, UNC-0638, on Sox2 protein accumulation. MCF7 cells were treated with the indicated doses of UNC-0638 for 24 h. Whole cell lysates were then harvested and analyzed by immunoblotting with G9a- and Sox2-specific antibodies.
(TIF)

S3 Fig The effect of chemical inhibition (BIX-01294) (left) or siRNA-mediated knockdown of G9a expression (right) on the amount of Sox2 protein in MDA-MB-231 cells was analyzed by immunoblotting. (TIF)

S4 Fig Immunoblot analysis of G9a expression levels in the MCF7, ZR-75-1, MDA-MB231, U-87MG, and T98G cells. β-actin was used as a loading control.
(TIF)

S5 Fig The transfection efficiency was analyzed by the detection of fluorescence 48 hrs after transfection of G9a-GFP expressing plasmid into MCF7 cells. (TIF)

S6 Fig The effect of G9a overexpression on the proliferation of MCF7 cells. Cell growth was analyzed by counting cell numbers at the indicated times after transfection with control or G9a plasmids. Abbreviations: Parent, wild-type MCF7 cells; C, MCF7 cells transfected with control plasmid; OE, MCF7 cells transfected with the G9a-expressing plasmid. *P < 0.05 compared with the control group. Data are expressed as the mean ± SD and are representative of three independent experiments.
(TIF)

S7 Fig The effect of G9a overexpression on the proliferation of MDA-MB-231 (A) and mouse ESCs (B). Cell growth was analyzed by counting cells at the indicated times post-BIX-01294 treatment. *P < 0.05 compared with the control group. Data are expressed as the mean ± SD and are representative of three independent experiments.
(TIF)

S1 Table Real-time RT-PCR primers. (DOCX)
