# Supplementary figures and images for: Novel Function of Lysine Methyltransferase G9a in the Regulation of Sox2 Protein Stability
Source: PLoS One. 2015 Oct 22;10(10):e0141118. doi: 10.1371/journal.pone.0141118 (PMC4619656; doi:10.1371/journal.pone.0141118)

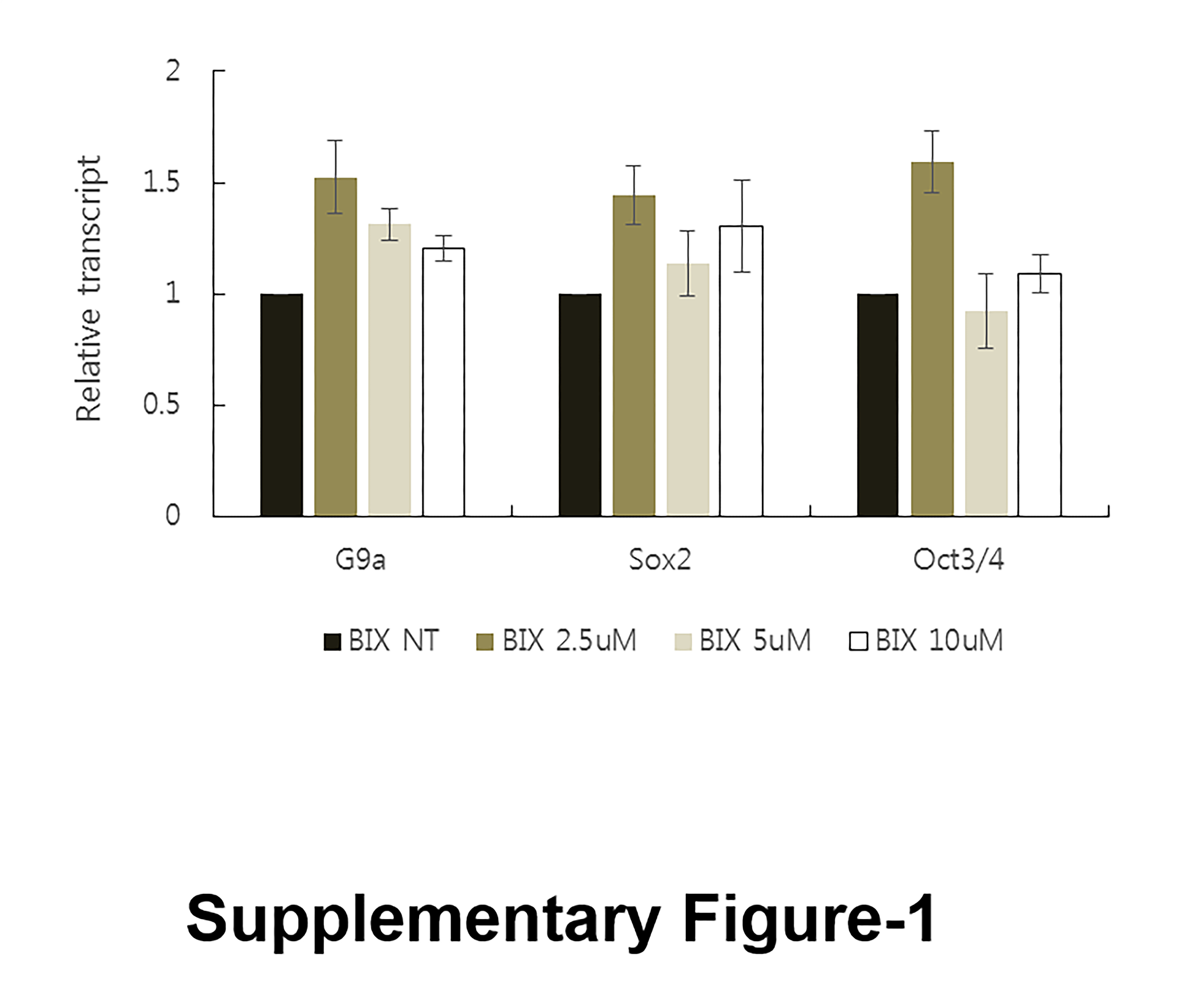

Supplement: S1 Fig — (TIF) [file pone.0141118.s001.tif]

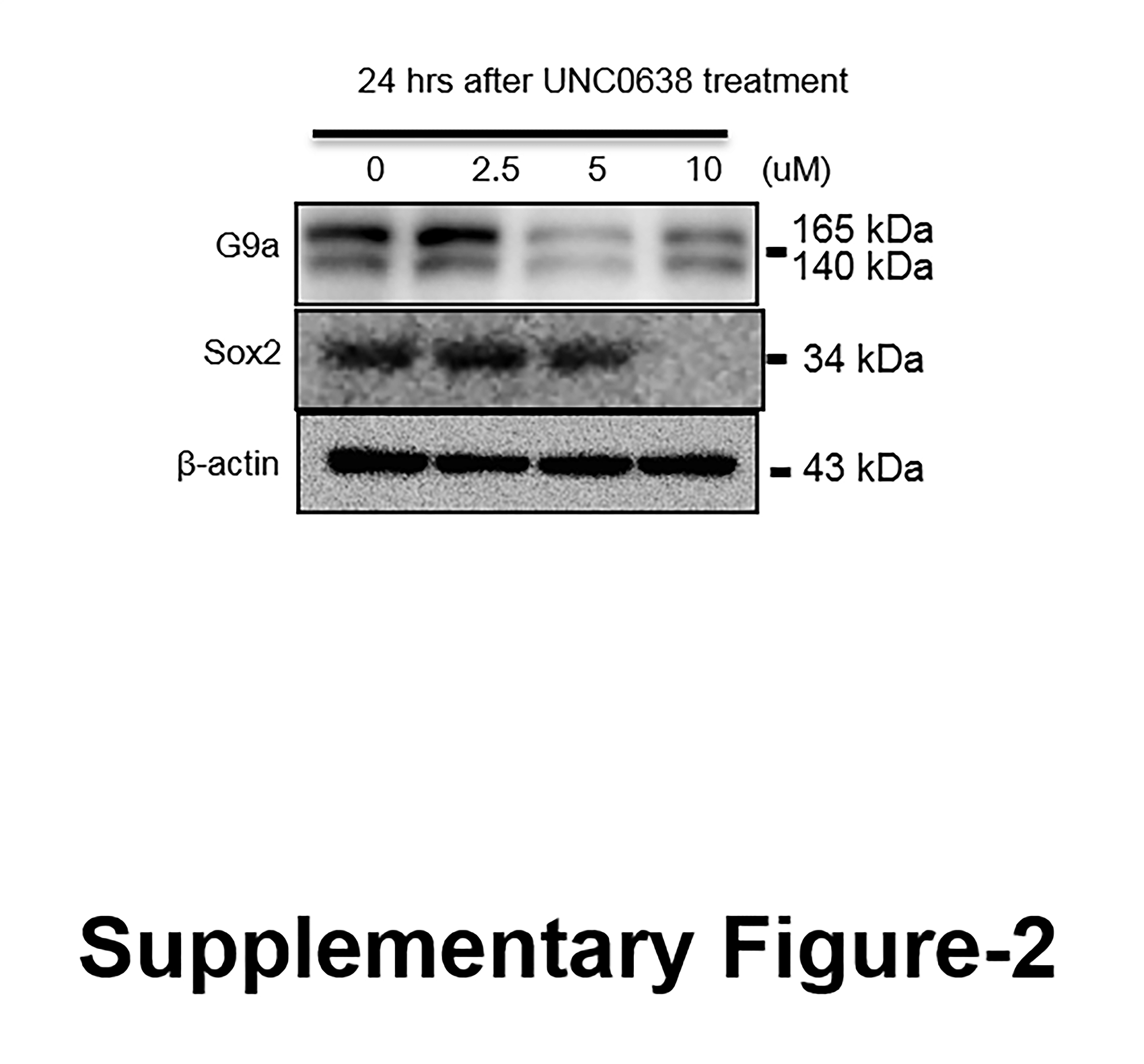

Supplement: S2 Fig — MCF7 cells were treated with the indicated doses of UNC-0638 for 24 h. Whole cell lysates were then harvested and analyzed by immunoblotting with G9a- and Sox2-specific antibodies. (TIF) [file pone.0141118.s002.tif]

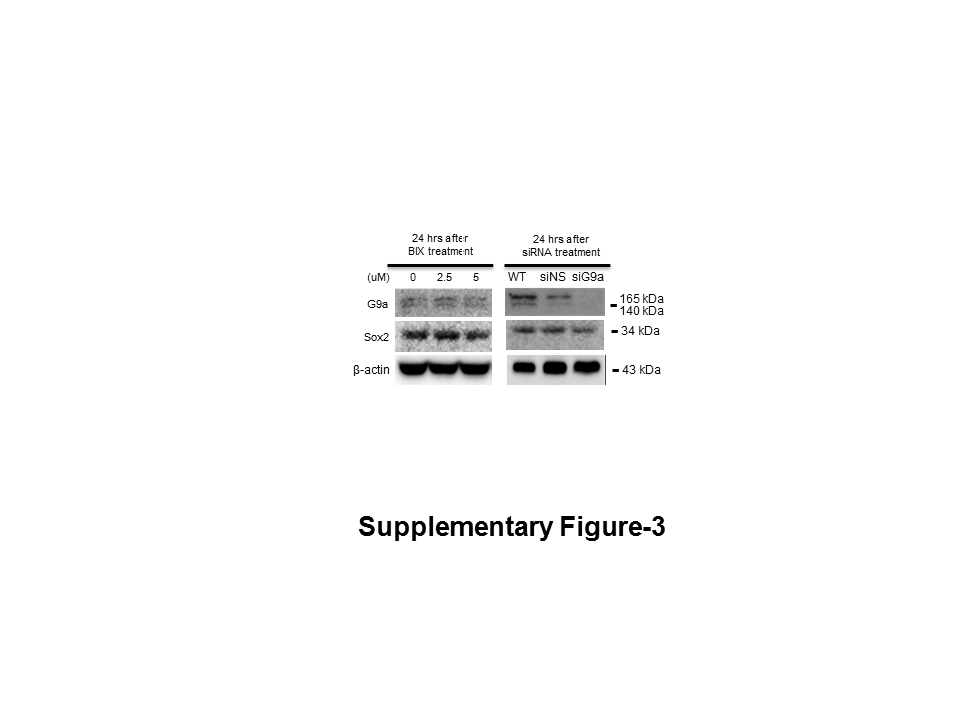

Supplement: S3 Fig — (TIF) [file pone.0141118.s003.tif]

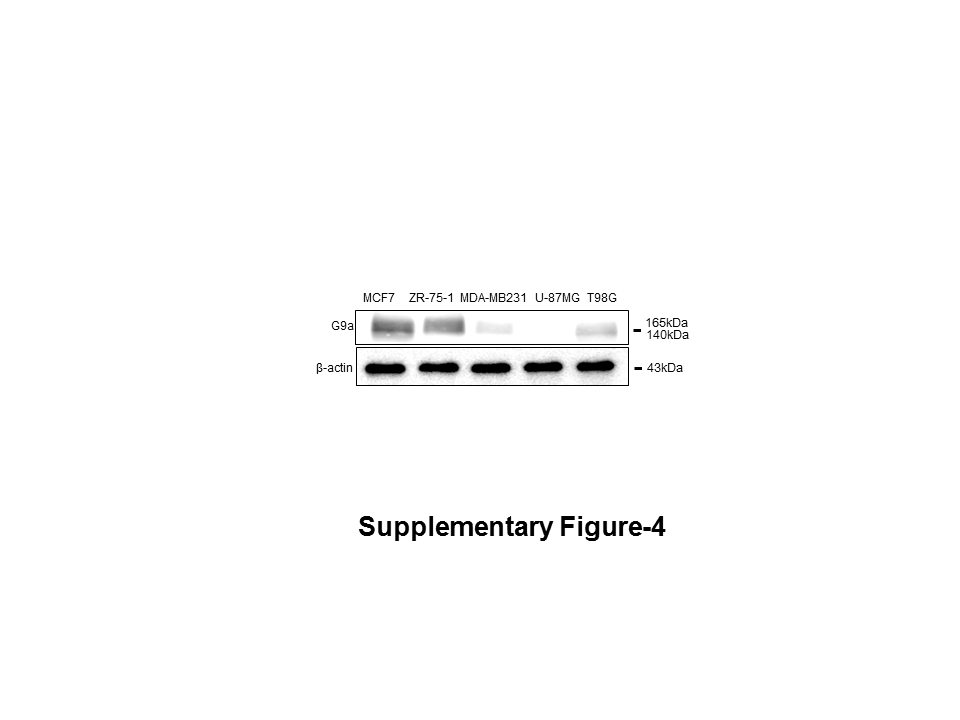

Supplement: S4 Fig — β-actin was used as a loading control. (TIF) [file pone.0141118.s004.tif]

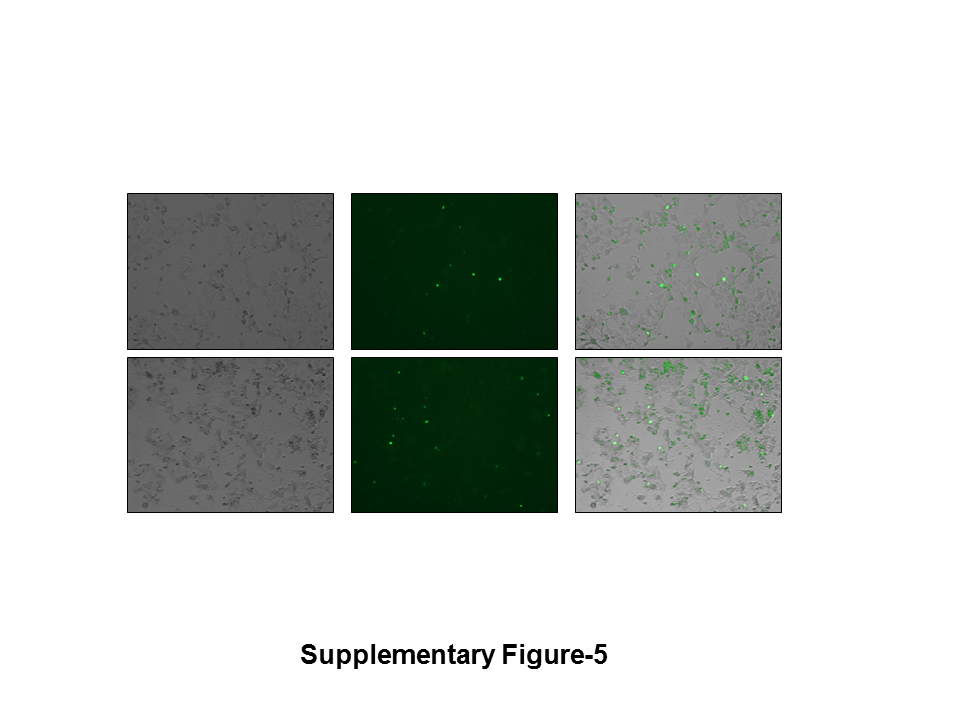

Supplement: S5 Fig — (TIF) [file pone.0141118.s005.tif]

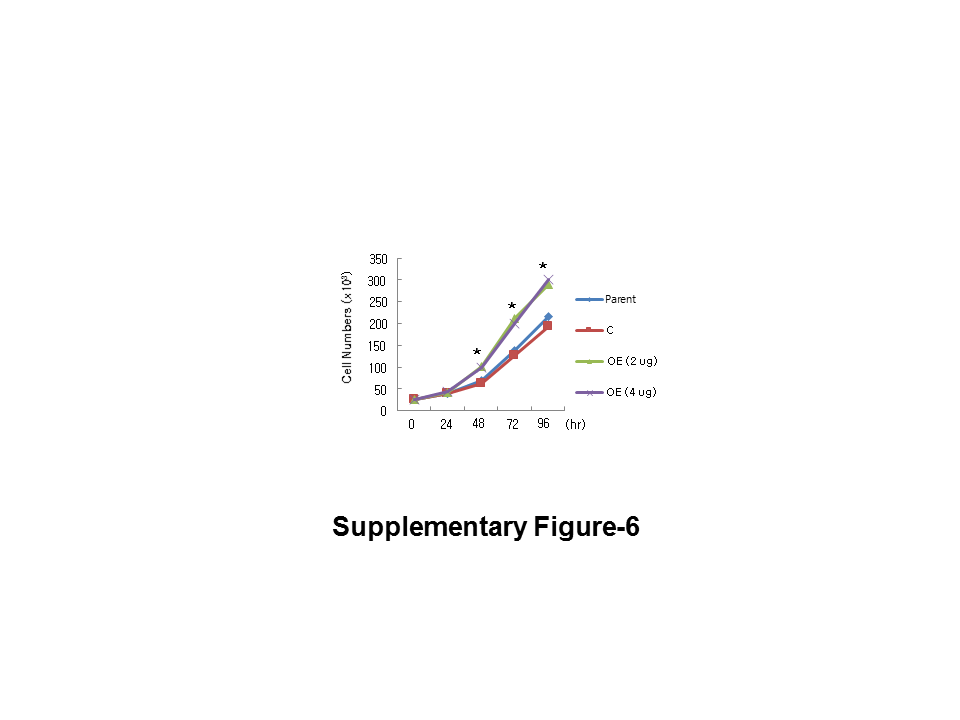

Supplement: S6 Fig — Cell growth was analyzed by counting cell numbers at the indicated times after transfection with control or G9a plasmids. Abbreviations: Parent, wild-type MCF7 cells; C, MCF7 cells transfected with control plasmid; OE, MCF7 cells transfected with the G9a-expressing plasmid. *P < 0.05 compared with the control group. Data are expressed as the mean ± SD and are representative of three independent experiments. (TIF) [file pone.0141118.s006.tif]

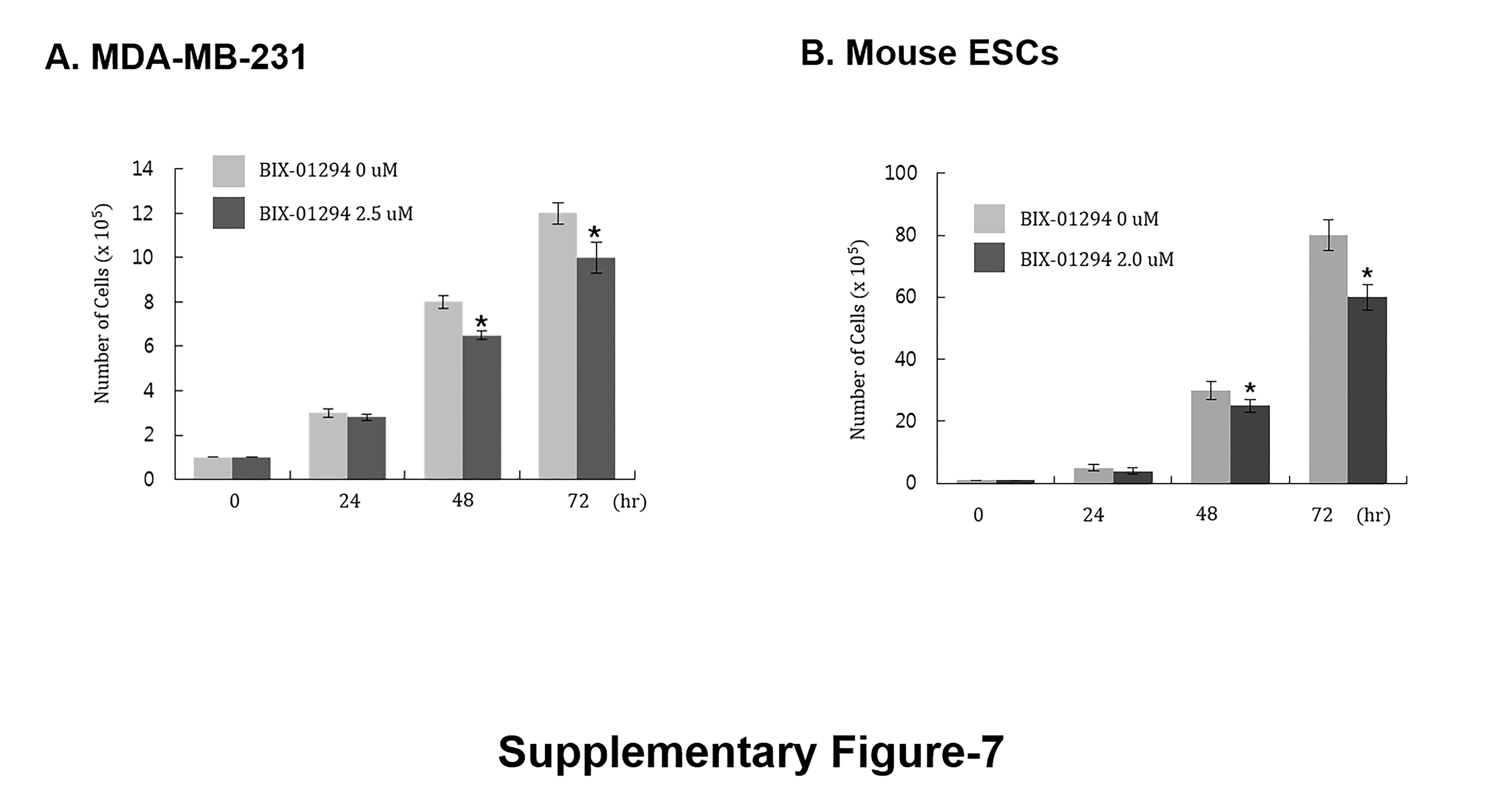

Supplement: S7 Fig — Cell growth was analyzed by counting cells at the indicated times post-BIX-01294 treatment. *P < 0.05 compared with the control group. Data are expressed as the mean ± SD and are representative of three independent experiments. (TIF) [file pone.0141118.s007.tif]
