# Supplementary material for: Novel Function of Lysine Methyltransferase G9a in the Regulation of Sox2 Protein Stability
Source: PLoS One. 2015 Oct 22;10(10):e0141118. doi: 10.1371/journal.pone.0141118 (PMC4619656; doi:10.1371/journal.pone.0141118)
Supplement: S1 Table — (DOCX) [file pone.0141118.s008.docx]

**Table S1.**

| Gene | Primer sequence (5’ to 3’) |
| --- | --- |
| GAPDH | F-acc aca gtc cat gcc atc ac |
|  | R-tcc acc acc ctg ttg ctg ta |
| SOX2 | F-tgg agt ggg agg aag agg ta |
|  | R-acc agc tcg cag acct ac at |
| G9aI | F-aaa atc ggg aac ttg gag a |
|  | R-ctc gtt gtc agt gag ggt ga |
| G9aII | F-agg ggt gtc caa tga cac at |
|  | R-tct cac aga gca cca tca gg |
| Oct4 | F-agc aaa acc cgg agg agt |
|  | R-cca cat cgg cct gtg tat atc |
